# FRETting about CRISPR-Cas Assays: Dual-Channel Reporting Lowers Detection Limits and Times-to-Result

**DOI:** 10.1021/acssensors.4c00652

**Published:** 2024-07-09

**Authors:** Jake M. Lesinski, Nathan K. Khosla, Carolina Paganini, Bo Verberckmoes, Heleen Vermandere, Andrew J. deMello, Daniel A. Richards

**Affiliations:** † Institute for Chemical and Bioengineering, ETH Zurich, Vladimir-Prelog-Weg 1, 8093 Zürich, Switzerland; ‡ Faculty of Medicine and Health Sciences, Department of Public Health and Primary Care, Ghent University, De Pintelaan 185, 9000 Gent, Belgium

**Keywords:** CRISPR, CRISPR-Cas, Cas12a, FRET, diagnostic, HPV-16, biosensors, molecular diagnostics, *cis* cleavage kinetics

## Abstract

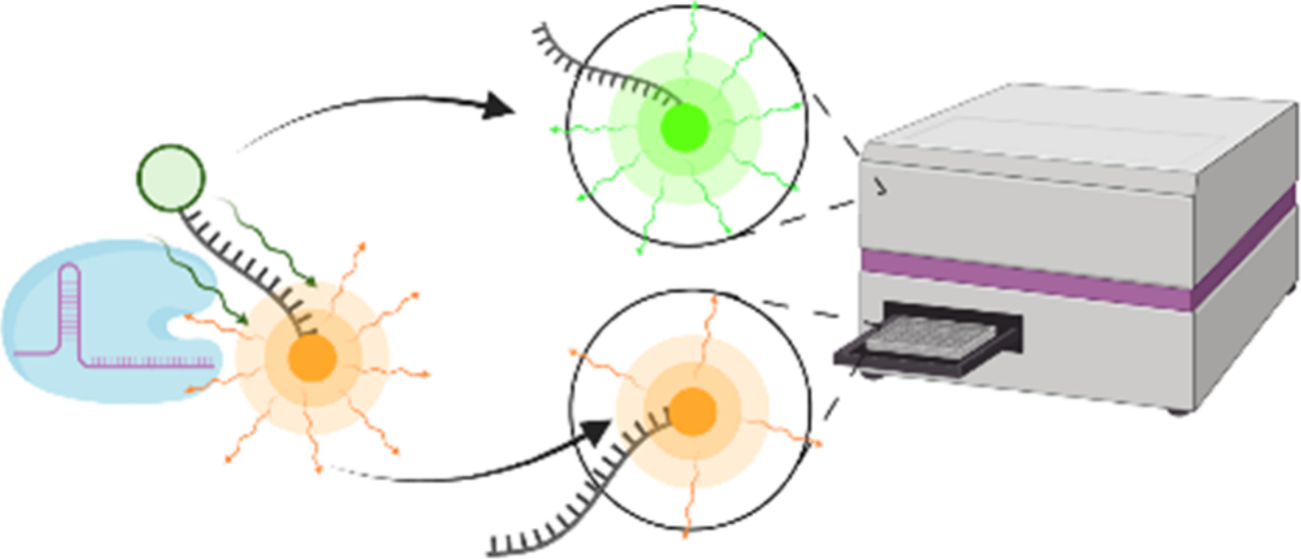

Clustered Regularly Interspaced Short Palindromic Repeats-CRISPR-Associated Protein (CRISPR-Cas) systems have evolved several mechanisms to specifically target foreign DNA. These properties have made them attractive as biosensors. The primary drawback associated with contemporary CRISPR-Cas biosensors is their weak signaling capacity, which is typically compensated for by coupling the CRISPR-Cas systems to nucleic acid amplification. An alternative strategy to improve signaling capacity is to engineer the reporter, i.e., design new signal-generating substrates for Cas proteins. Unfortunately, due to their reliance on custom synthesis, most of these engineered reporter substrates are inaccessible to many researchers. Herein, we investigate a substrate based on a fluorescein (FAM)–tetramethylrhodamine (TAMRA) Förster resonant energy-transfer (FRET) pair that functions as a seamless “drop-in” replacement for existing reporters, without the need to change any other aspect of a CRISPR-Cas12a-based assay. The reporter is readily available and employs FRET to produce two signals upon cleavage by Cas12a. The use of both signals in a ratiometric manner provides for improved assay performance and a decreased time-to-result for several CRISPR-Cas12a assays when compared to a traditional FAM–Black Hole Quencher (BHQ) quench-based reporter. We comprehensively characterize this reporter to better understand the reasons for the improved signaling capacity and benchmark it against the current standard CRISPR-Cas reporter. Finally, to showcase the real-world utility of the reporter, we employ it in a Recombinase Polymerase Amplification (RPA)–CRISPR-Cas12a DNA Endonuclease-Targeted CRISPR Trans Reporter (DETECTR) assay to detect *Human papillomavirus* in patient-derived samples.

With increasing globalization, and the accompanying rise in the spread of infectious diseases, the importance of rapid, accessible, sensitive, and specific nucleic acid testing has become increasingly apparent.^1^ To this end, a plethora of nucleic acid amplification tests (NAATs) have been developed to diagnose and monitor the spread of disease, improve patient treatment, and decrease socioeconomic burdens.^2^ The field of molecular diagnostics is currently dominated by such NAATs, particularly the polymerase chain reaction (PCR). First introduced in 1985, PCR remains the most regularly employed amplification technique in nucleic acid amplification tests.^3^ However, despite nearly four decades of optimization and development, the diagnostic utility of PCR is still hindered by several limitations, including cost, time-to-result, and a reliance on thermocycling.^2^ Understanding these limitations, researchers have begun to adopt alternative methods for detecting nucleic acids, including isothermal NAATs and, more recently, Clustered Regularly Interspaced Short Palindromic Repeats-CRISPR-Associated Protein (CRISPR-Cas)-based diagnostics.^2,4^ CRISPR-Cas technologies were initially developed for gene editing applications, primarily using Cas9.^5^ Nevertheless, recent research has demonstrated the potential of other CRISPR-associated proteins, such as those in the Cas12, Cas13, and Cas14 families, to act as biosensors for disease diagnostics.^6^ These lesser-known Cas enzymes act as molecular “IF” operators, unleashing a signal cascade if a target nucleic acid is present.^7^ Specifically, if a target nucleic acid binds to a single-guide RNA (sgRNA) via Watson–Crick base pairing, cleavage of the target nucleic acid occurs (*cis*, or “targeted”, cleavage). This is followed by a conformational change in the Cas protein that allows it to engage in nontargeted cleavage (*trans*, or “collateral”, cleavage) of single-stranded DNA (ssDNA). Such collateral cleavage can then be leveraged to generate a signal. This is typically achieved by adding ssDNA reporters that contain a fluorophore on one end and a quencher on the other.^7^ This basic idea underpins a large number of CRISPR-Cas platforms, including the Specific High-sensitivity Enzymatic Reporter Unlocking (SHERLOCK)^8^ and DNA Endonuclease-Targeted CRISPR Trans Reporter (DETECTR)^7^ assays. These systems utilize isothermal NAATs in combination with CRISPR assays to enhance the signaling capacity of CRISPR-Cas systems while simultaneously exploiting their exquisite target specificity.^7−13^ Seeing the potential of CRISPR-Cas systems, researchers have subsequently reported new assay platforms (e.g., one-pot NAAT–CRISPR^8,11^ and droplet-based CRISPR-Cas assays^14^), novel Cas12/13 variants (e.g., thermostable),^15,16^ engineered reporter substrates,^17^ or different sgRNA formats (such as multiple sgRNAs for a single target).^18^ These modifications typically lead to lower limits-of-detection (LoDs), greater analytical sensitivity, reduced time-to-result, or improved assay practicality and robustness. An excellent overview of recent advances is provided by Weng et al.^19^

Of particular relevance to the current work is the exploration of different fluorescence reporters for CRISPR-Cas12 assays. The seminal paper introducing DETECTR employed a fluorescent reporter based on a TTATT ssDNA sequence that could be effectively cleaved by Cas12a, leading to a fluorescent signal.^7,20^ While this sequence was universally adopted for several years, recent reports have demonstrated that Cas12a has a preference for cleaving polycytosine chains; hence, the use of a reporter based on a hexameric chain of cytosine (C) residues is now gaining traction.^14,21^ Additionally, the vast majority of the fluorescence-based CRISPR-Cas formats utilize dark quenchers, such as Black Hole quencher (BHQ) or Iowa Dark quencher dyes.^7,22^ Here, a dark quencher dye absorbs excitation energy from a nearby fluorophore via Förster resonant energy transfer (FRET), dissipating this energy as heat and generating a very low fluorescence background signal. While useful in CRISPR-Cas formats, dark quenchers, such as BHQ, are highly susceptible to degradation from other assay components, most notably reducing agents such as dithiothreitol (DTT).^23^ Alternative nonsmall molecule FRET reporters have been noted.^24,25^ However, surprisingly little attention has been paid to the use of conventional FRET-based systems, where a donor fluorophore transfers energy to an acceptor fluorophore (through nonradiative dipole–dipole coupling), with the acceptor fluorophore then dissipating energy in a radiative manner. Whereas a single study by Liu and co-workers demonstrated a fluorescence FRET probe using fluorescein (FAM) and tetramethylrhodamine (TAMRA), no work has been done to show drop-in integration of such a reporter into state-of-the-art assay chemistries or elucidating the reasons behind the ratiometric reporter’s better performance.^26^ Accordingly, and to further investigate the potential of FRET-based reporters in CRISPR-Cas systems, we herein optimized a CRISPR-Cas reporter system that utilizes a FAM–TAMRA FRET pair (Figure 1). Within the CRISPR-Cas system, activated Cas12a will cleave the probe, resulting in a decrease in the TAMRA fluorescence (at 583 nm) and an increase in FAM emission at 530 nm. Interestingly, this reporter displays faster cleavage rates than its BHQ-based counterpart and is significantly more resistant to common additives required for efficient CRISPR-Cas12a-based assays. Moreover, by exploiting the ratio between “green” and “red” fluorescence of the dual-reporter, significant enhancements in signal-to-noise ratio when compared to BHQ-based reporters are achieved. Finally, and to demonstrate the broad utility and compatibility of this reporter in existing diagnostic formats, we implement the reporter in several model CRISPR-Cas12a-based assays, observing improved sensitivities and reduced times-to-result. Ultimately, this study highlights the potential of visible FRET-based reporters as “drop-in” replacements for conventional dark quencher-based reporters in CRISPR-Cas-based biosensors.

**Figure 1:**
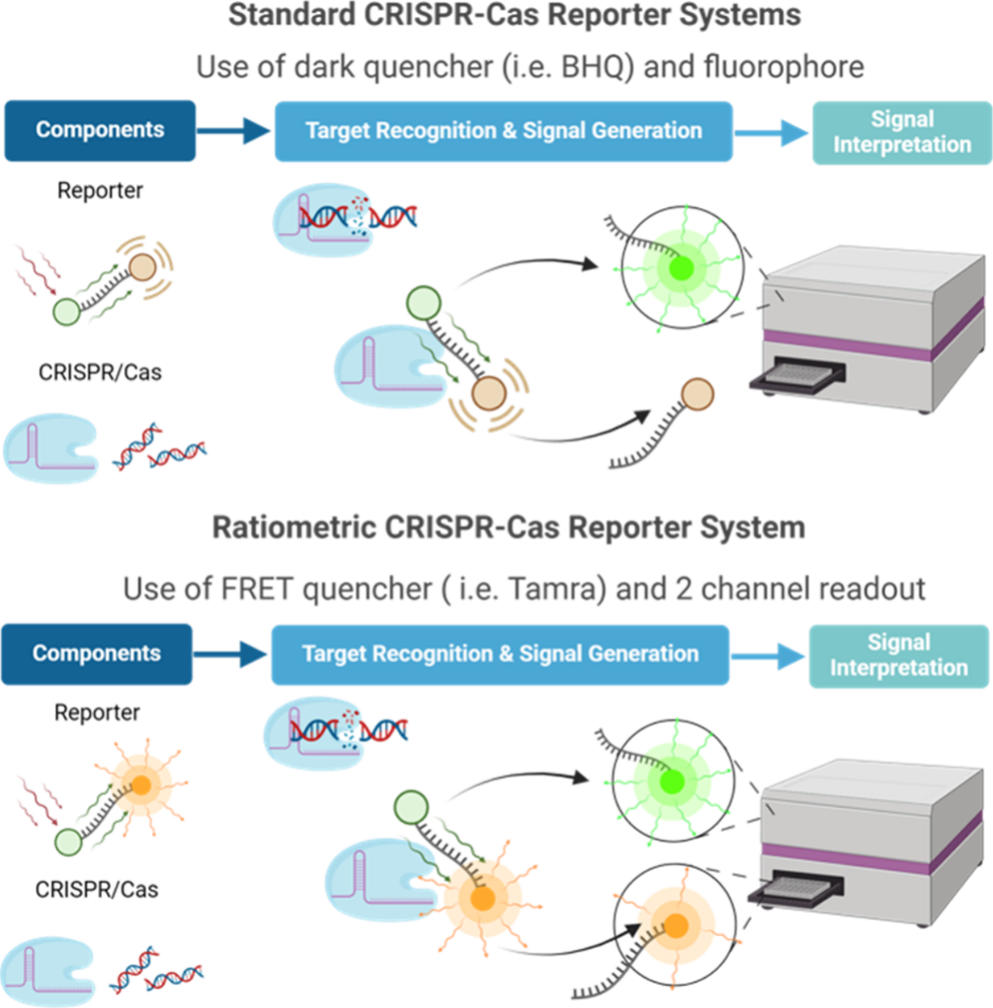
Ratiometric CRISPR-Cas12a system. Schematic representation of the dual-channel FRET-based CRISPR-Cas12a reporter system. Current (Top) reporter systems rely on a fluorophore-quench reporter comprising a dark (nonradiative) quench to prevent emission and a single-channel readout to detect cleaved reporter. The proposed readout system instead uses a visible-light quench. While the reporting fluorophore remains bound to the quencher, the reporter system forms a FRET pair and emits light in the emission wavelength of the visible quench. Upon cleavage by Cas12a, the signal in the reporting fluorophore emission channel increases, while the signal in the quench emission channel decreases. The analyzed signal is thus the ratio of the increasing reporting fluorophore emission channel to the decreasing quench emission channel.

## Experimental Section

### Evaluating BHQ1 and TAMRA in DTT-Containing Buffer

To solutions of either FAM–CCCCCC–BHQ-1 or FAM–CCCCCC–TAMRA (70 μL, 1 μM, 2× HOLMES buffer), dithiothreitol (70 μL, 2–20 mM, UltraPure water) or UltraPure water (negative control) was added. Samples were spit into aliquots (5 × 20 μL) and added to a 384 black well plate (Corning). Mineral oil (2.5 μL) was added to each well and the plate centrifuged for 1 min (1000 r.c.f.). The plate was then placed into a plate reader (BioTek Synergy H1) and the emission measured (Ex_484_/Em_530_) every 2 min for 180 min.

### Evaluating the Kinetics of CRISPR-Cas12a with BHQ and TAMARA

To a solution of LbCas12a (200 μL, 230 nM, 1× HOLMES buffer) and HPV-16 crRNA (200 μL, 230 nM, 1× HOLMES buffer) was added. To this solution, HPV-16 activating DNA (1 μL, 100 pM, UltraPure water) was added, and the solution was incubated for 30 min at 37 °C to create the Cas12a complex. The resulting complex (5 μL) was mixed with either the FAM–CCCCCC–BHQ-1 or FAM–CCCCCC–TAMRA reporter (17 μL, 1.29–2588 nM) in triplicate, pipet mixed, then added to a chilled 384 black well plate (Corning), which was kept on ice. Mineral oil (2.5 μL) was added to each well and the plate centrifuged for 1 min (1000 r.c.f.). The plate was then placed into a plate reader (BioTek Synergy H1) and the emission measured (Ex_484_/Em_530_) every 2 min for 180 min.

### CRISPR-Cas12a Single-Guide Assay with Synthetic Activating DNA

To a solution of LbCas12a (217.5 μL, 115 nM, 1× HOLMES buffer), HPV-16 crRNA (2.5 μL, 10 μM, UltraPure water) was added and the solution incubated at 37 °C for 30 min to produce the Cas–RNA complex. This solution was aliquoted (5 μL), and to each aliquot synthetic target DNA (1 μL, varying concentrations, UltraPure water), FAM–CCCCCC–BHQ-1 or FAM–CCCCCC–TAMRA reporter (0.23 μL, 50 μM, UltraPure water), 10× HOLMES buffer (2.3 μL), and UltraPure water (14.47 μL) were added. These solutions were prepared in triplicate for each target DNA concentration. The samples were pipet mixed, then added to a chilled well 384 black well plate (Corning), which was kept on ice. Mineral oil (2.5 μL) was added to each well and the plate centrifuged for 1 min (1000 r.c.f.). The plate was then placed into a plate reader (BioTek Synergy H1) and the emission measured (Ex_484_/Em_530_ and Em_583_) every 2 min for 180 min.

### CRISPR-Cas12a Double-Guide Assay with Synthetic Activating DNA

To two separate solutions of LbCas12a (217.5 μL, 115 nM, 1× HOLMES buffer), HPV-16 crRNA1 or HPV-16 cRNA2 (2.5 μL, 10 μM, UltraPure water) was added and the solution incubated at 37 °C for 30 min to produce the two Cas–RNA complexes. These two complexes were utilized in the same manner as described above in *CRISPR-Cas12a Single-Guide Assay with Synthetic Activating DNA*.

### CRISPR-Cas12a DETECTR Assay with Clinical Samples

The assay utilized for analyzing the clinical samples described above was directly reproduced from Chen et al.^7^Recombinase Polymerase Amplification (RPA) was performed on all samples using the Twist Basic RPA kit according to the manufacturer’s guidelines. Briefly, to a solution of rehydration buffer (29.5 μL), UltraPure water (11.2 μL), forward and reverse primers (2.4 μL each, 10 μM, UltraPure water), magnesium acetate (2.5 μL, 280 mM, UltraPure water), and the respective clinical sample (2 μL) were added. The solutions were incubated at 37 °C for 10 min and then placed into an ice slurry to quench the reaction. Each amplified clinical sample (1 μL) was then analyzed using the optimized CRISPR-Cas12a assays, with both FAM–CCCCCC–BHQ and FAM–CCCCCC–TAMRA reporters, as described above.

### Analysis of Ratiometric Signal

To enable ratiometric readout, CRISPR-Cas12a-based assays were performed as previously described, but instead of using a single channel (530 ± 9 nm) to measure FAM emission only, we employed two detection channels (530 ± 9 nm and 583 ± 9 nm for FAM and TAMRA, respectively) to generate a ratiometric signal. The FRET quotient, *Q*, is given by
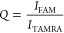
1Where *I*_FAM_ is the FAM channel intensity and *I*_TAMRA_ is the TAMRA channel fluorescence intensity. Further information regarding propagating errors through the quotient calculation can be found in the SI (Figure S2).

## Results and Discussion

### Comparison of BHQ-1- and TAMRA-Based Reporters in a CRISPR-Cas12a Assay

**Figure 2:**
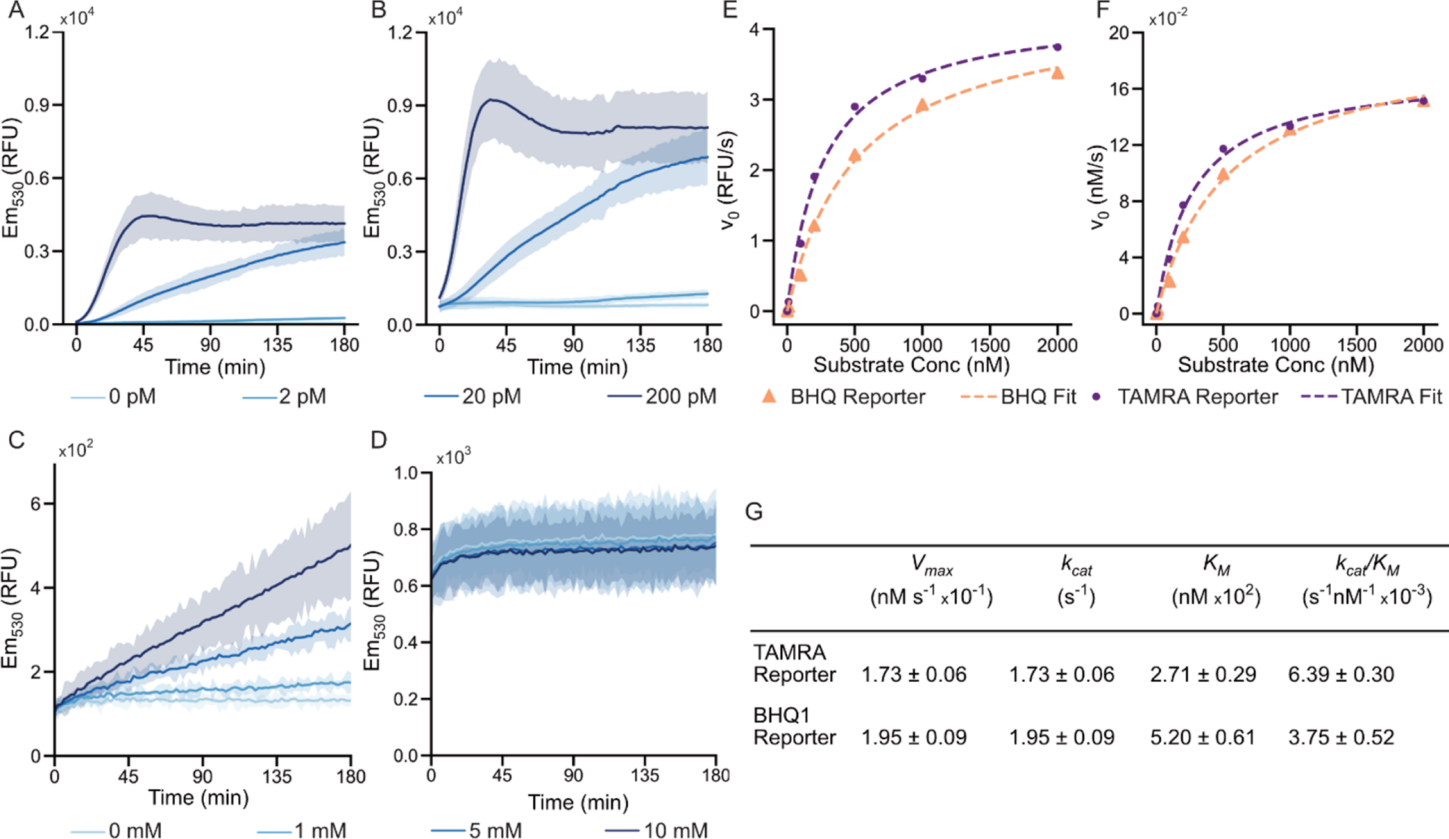
Comparison and degradation evaluation of both TAMRA and BHQ-based reporters. Single-channel (Ex_484_/Em_530_) fluorescence readout from a standard CRISPR-Cas12a assay using (A) a standard FAM–CCCCCC–BHQ1 reporter and (B) the FAM–CCCCCC–TAMRA reporter. (C) Fluorescence readout from incubation of the FAM–CCCCCC–BHQ1 reporter in buffers with varying DTT concentrations. Whereas drift in the low-DTT-containing buffers is minimal, there is significant drift in the higher concentration as DTT degrades BHQ1, thus impeding quenching efficiency. (D) Fluorescence readout from incubation of the FAM–CCCCCC–TAMRA reporter in buffers with varying DTT concentration. Here, drift is detectable at all concentrations. (E) Michaelis–Menten fit of initial velocity in RFU/s at varying concentrations of both reporters. (F) Michaelis–Menten fit of the initial velocity at varying concentrations of both reporters in nM/s. The conversion from RFU to nM was made using an internal calibration using the plateaus of the 0, 10, 100, and 200 nM reporter curves (Figure S3). (G) Michaelis–Menten kinetic parameters for LbCas12a cleavage of both BHQ1 and TAMRA-based reporters. While the TAMRA-based reporter has a slightly lower *k*_cat_ it also has a drastically lower *K*_M_ leading to a *k*_cat_*/K*_M_ roughly double that of the BHQ1-based reporter. All plots with error shades show the mean ±3 standard deviations.

To evaluate the potential of the FRET-based reporter as a fluorometric probe for CRISPR-Cas12a assays, we utilized a 5-Carboxytetramethylrhodamine (TAMRA)-based reporter (5′-FAM-CCCCCC-TAMRA-3′) and benchmarked it against a conventional BHQ1-based equivalent (5′-FAM-CCCCCC-BHQ1–3′). TAMRA was chosen as the acceptor due to its excellent spectral overlap with FAM and the fact that it has previously been validated as a FAM partner in ratiometric probes.^26^ The poly-C linker was chosen based on its advantageous cleavage kinetics over other short sequences.^14,21^ Due to its extensive prior use in CRISPR-Cas diagnostic assays, we selected the hypervariable loop V of the L1-encoding gene of *Human papillomavirus 16* (HPV16) as our DNA target.^7^ HPV is a common sexually transmitted infection, and HPV16 in particular is strongly associated with an increased risk of cervical cancer in women.^27^ As an initial test, we performed an amplification-free CRISPR-Cas12a-based assay using both reporters across a range of target concentrations (Figure 2A,B). Noting the significantly greater signals obtained using the TAMRA-based reporter, we next measured the *trans* cleavage rates of each reporter using a Cas12a-sgRNA complex preactivated with synthetic HPV-16 target DNA (Figures 2E,F and S3), by extracting the catalytic constants *k*_cat_ and *K*_M_ through Michaelis–Menten analysis (Figure 2G). Finally, and due to the known instability of BHQ in media containing DTT, we investigated the stability of both reporters in the assay buffer over time (Figure 2C,D).^28^

These data highlight several interesting trends. The TAMRA-based reporter yields a greater fluorescence signal over a shorter period of time than the BHQ-based reporter. When compared to the traditional BHQ-based reporter, the TAMRA-based reporter granted signal increases of 2.24, 1.85, and 1.87 times greater for target concentrations of 2, 20, and 200 pM, respectively. We attribute this to an enhanced capacity of Cas12a to cleave the reporter, as evidenced by the Michaelis–Menten analysis. We hypothesize that kinetic enhancements are due to the formation of molecular dimers. Although FRET pairs are conventionally designed by optimizing resonance energy transfer (through maximization of spectral overlap between donor emission and acceptor absorption), other mechanisms of energy transfer exist. For example, the BHQ family of quencher dyes is known to participate in static quenching with FAM, where reporter and quencher noncovalently associate into a molecular dimer, allowing a more direct coupling of energy levels and thus higher quenching efficiencies.^29,30^ While this effect actually serves to lower background fluorescence of the quenched reporter, it may also inhibit subsequent Cas12a collateral cleavage, since oligonucleotides with dimerized fluorophore and quench could be less accessible. In addition, the TAMRA-based reporter suffered relatively less background drift in an optimized CRISPR-Cas12a buffer containing DTT (Figure 2C,D). We attribute this observation to the increased stability of TAMRA in a reducing environment relative to the BHQ, which is known to degrade in the presence of strong reducing agents.^23^ Taken together, the combined effects of increased cleavage rates and higher stability make a compelling case for using TAMRA-based reporters in CRISPR-Cas assays rather than conventional dark quencher-based reporters.

### Ratiometric Versus Single-Channel Readout

**Figure 3:**
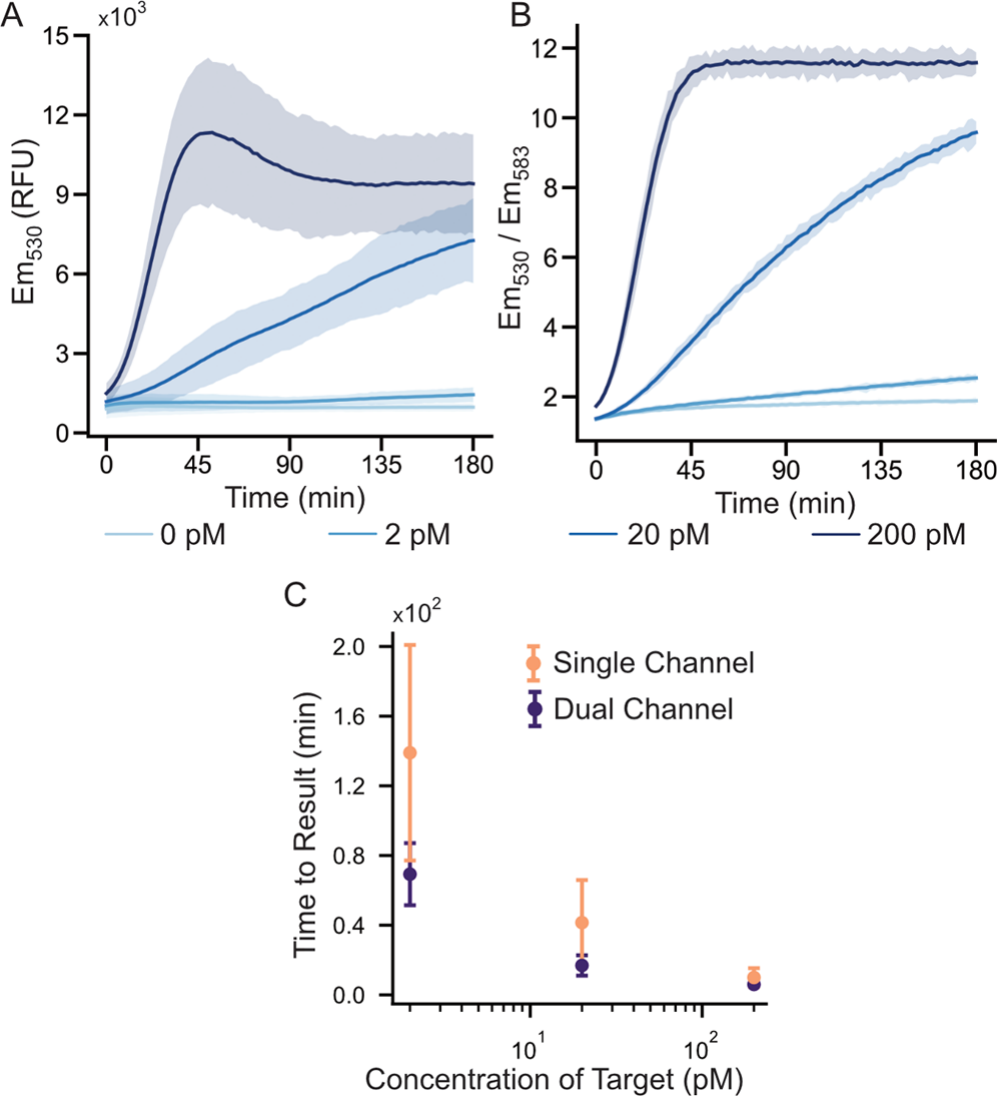
Comparison of single-channel and dual-channel ratiometric measurement methods. (A) Fluorescence readout from a standard CRISPR-Cas12a assay using the FAM–CCCCCC–TAMRA reporter. To ensure statistical significance, each concentration was done with *n* = 20. (B) The same assay as (A), but this time assessed with the emission measured in two channels (530 and 583 nm), with the data reported as the ratio of *I*_530_ to *I*_583_. (C) A time-to-significance (TTS) chart showing how long each sample titer took to become statistically different from the negative. Statistical significance is defined as the negative plus 3 standard deviations being lower than the positive minus 3 standard deviations. At all assay titers, the ratiometric measurement performs better, although the difference was significantly less at high concentrations. All plots with error shades show the mean ±3 standard deviations.0

After confirming the enhanced cleavage kinetics of the TAMRA-based reporter, we next investigated its utility as a ratiometric reporter, which have been shown to reduce both signal variations and artifacts in FRET-based studies.^31−33^ Although the implementation of ratiometric analysis is often used in cellular imaging to correct for experimental artifacts, local variations in fluorophore concentration, or cellular density, we employed it to correct for unavoidable stochastic processes in our CRISPR-Cas12a-based assays that can cause variations in fluorescent signal, through nonspecific adsorption or evaporation, for example. We compared single-channel FAM data (Figure 3A) to the ratiometric data (Figure 3B) to generate a time-to-significance (TTS) analysis (Figure 3C). The time-to-significance analysis compares a positive test with the corresponding negative control and finds the time, *t*, at which a given sample statistically escapes similarity with its corresponding average negative. A positive is deemed statistically significant when its 99.7% confidence interval does not overlap with the 99.7% confidence interval of the corresponding negative. Such an approach additionally eliminates false positives due to random variability.

In the data, we see a clear reduction in measurement uncertainty. This lower uncertainty in both positive and negative tests provides for a faster time-to-result, even at target concentrations as low as 2 pM. This directly translates into the ability to meaningfully detect lower concentrations of analyte at a given assay time, with the ratiometric readout able to differentiate between the 2 pM sample and the negative within 70 min, while single-channel readout took 139 min. Further, there is a significant change in the shape of the intensity versus time curve. This is most evident in the data for the 200 pM positive sample, where FAM fluorescence increases with time, reaches a maximum at 45 min, and then drops to a steady-state value after approximately 120 min (Figure 3A). No such effect is observed for the ratiometric analysis, as shown in Figure 3B. We posit that this aberration (and associated correction in the ratiometric measurement) is due to the adsorption of the reporting fluorophore to the well plate. In the single-channel measurement, fluorescence intensity decreases as both donor and acceptor adsorb to the well walls. Conversely, in a dual-channel setup, the ratiometric measurement remains constant as long as both the cleaved FAM and cleaved TAMRA adsorb at similar rates (Figure S4).

### Implementation of FRET-Based Reporters in Double-Guide CRISPR-Cas Assays

**Figure 4:**
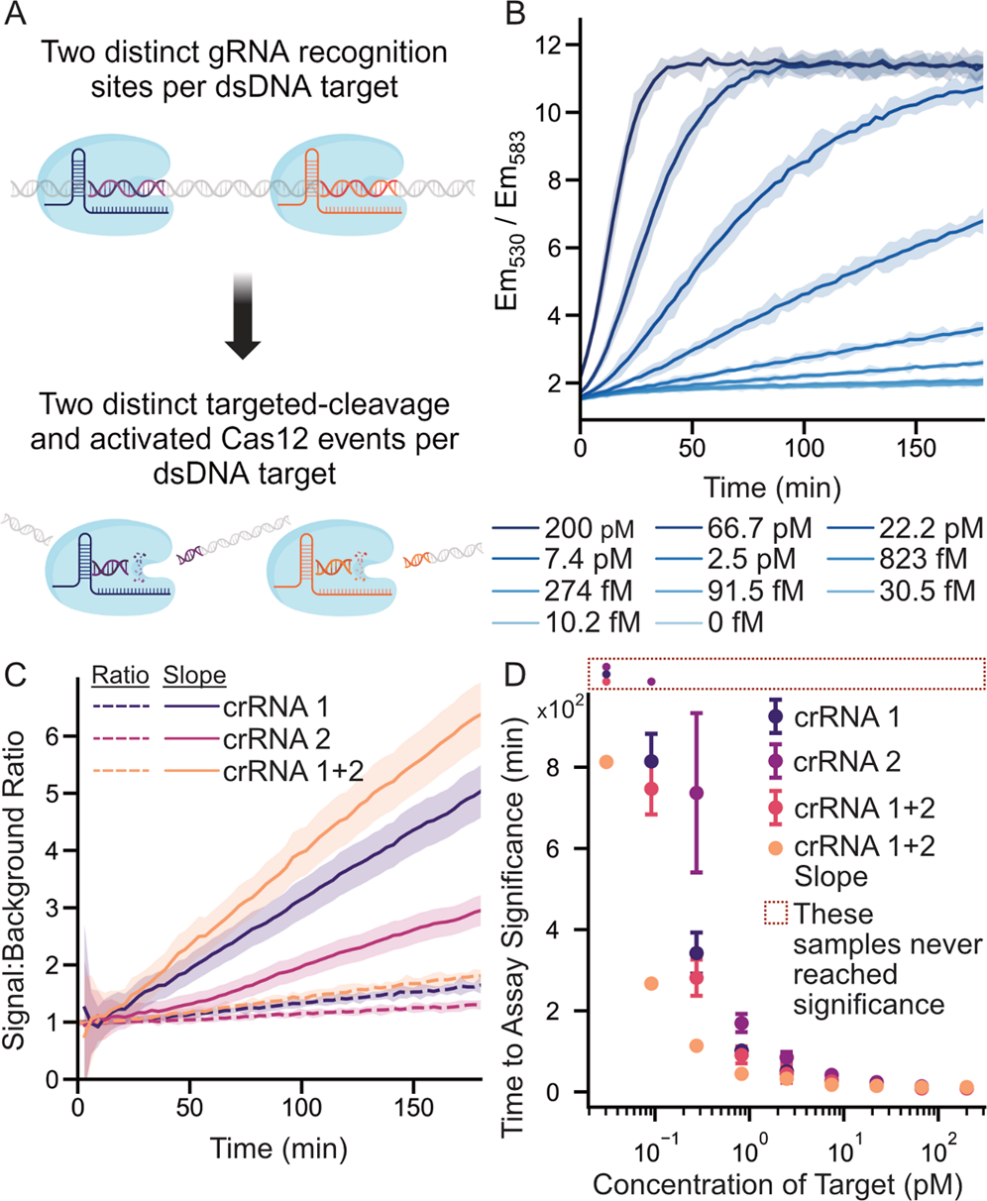
Integration of the ratiometric reporter with a double sgRNA assay. (A) A schematic of the double sgRNA system. Two different CRISPR-RNA complexes (using two different sgRNAs) allow a single target DNA to activate two CRISPR enzymes, thus (in theory) doubling the rate of signal generation. (B) Ratiometric readout of the double-guide assay. (C) Comparison of the two sgRNA guides and the combined guide assay. Signal-to-background analysis using both ratiometric fluorescence output as well as a slope algorithm. The *y* axis is normalized to a signal-to-background ratio by dividing the signal by its corresponding negative at each time. All assays were performed using 2 pM of trigger DNA. (D) A time-to-significance plot showing how each sgRNA performs, along with a slope-based analysis of the dual-guide assay. The data demonstrate the potential of the ratiometric reporter to act as a “drop-in” replacement in CRISPR-Cas12 assays. All plots with error shades show the mean ±3 standard deviations.

A key feature of the FRET-based reporter is that it can replace traditional dark-quench reporters in common CRISPR-Cas assays without requiring any alteration of assay reagents or conditions. To validate this “drop-in” feature, we used the FRET-based reporter in a dual-sgRNA-based assay, which has previously been shown to reduce time-to-result compared to sgRNA assays.^18^ Following the procedure reported by Fozouni et al.,^18^ we complexed two separate solutions of Cas12a enzyme with two different sgRNAs, each targeting a different section of an amplicon on the L1-encoding gene of HPV16 (Figure 4A). We then performed an amplification-free CRISPR-Cas12-based assays at multiple target DNA concentrations (Figure 4B). Both enzymes were also characterized individually in a single-guide assay (Figure S5). To enable comparison between both single- and dual-guide assays, we calculated the signal-to-background ratio for each assay by dividing the signal at any given time by the corresponding signal in the negative assay. A similar analysis was also performed using a slope algorithm, in which the slope of fluorescence intensity vs time at a given assay time point, *t*, is calculated as the linear regression from *t = 0* to *t*. This method has been previously used to decrease time-to-result in CRISPR-Cas12a-based assays.^18^ The signal-to-background analysis, including both the end point fluorescence and slope analysis for a target concentration of 2 pM, is shown in Figure 4C. As a further comparison, assays employing the two individual sgRNAs, as well as the dual sgRNA and slope analysis, were compared in terms of the time-to-significance (Figure 4D).

These data mirrored the trend found in the single-guide assay, i.e., sgRNA 1 was faster than sgRNA 2 (faster time-to-result), with the slope analysis greatly decreasing the time-to-result. These data confirm the capacity of ratiometric reporters to decrease the limit-of-detection and time-to-result when compared to conventional BHQ-based reporters while requiring no change in assay conditions. While the use of slope analysis affords improvements in performance, it introduces considerable noise at early assay times. This is due to the fact that at early times the slope is estimated from a limited number (as few as two) of measurements. Whereas a pure end point fluorescence may identify high titer samples within only a few readings, a slope-based approach may have a higher minimum test time no matter the titer. Interestingly, we found that our double-guide system (sgRNA 1 + sgRNA 2) led to only minimal performance improvements when compared to our single-guide (sgRNA 1) system. We attribute this to the relatively poor performance of sgRNA 2, which was not able to add significantly to the signals attributed to sgRNA 1. Though previous work has shown that the double-guide assays can significantly outperform single-guide assays, this work also employed sgRNAs that performed similarly when measured individually.^18^

### Application to Patient-Derived HPV16 Samples

**Figure 5:**
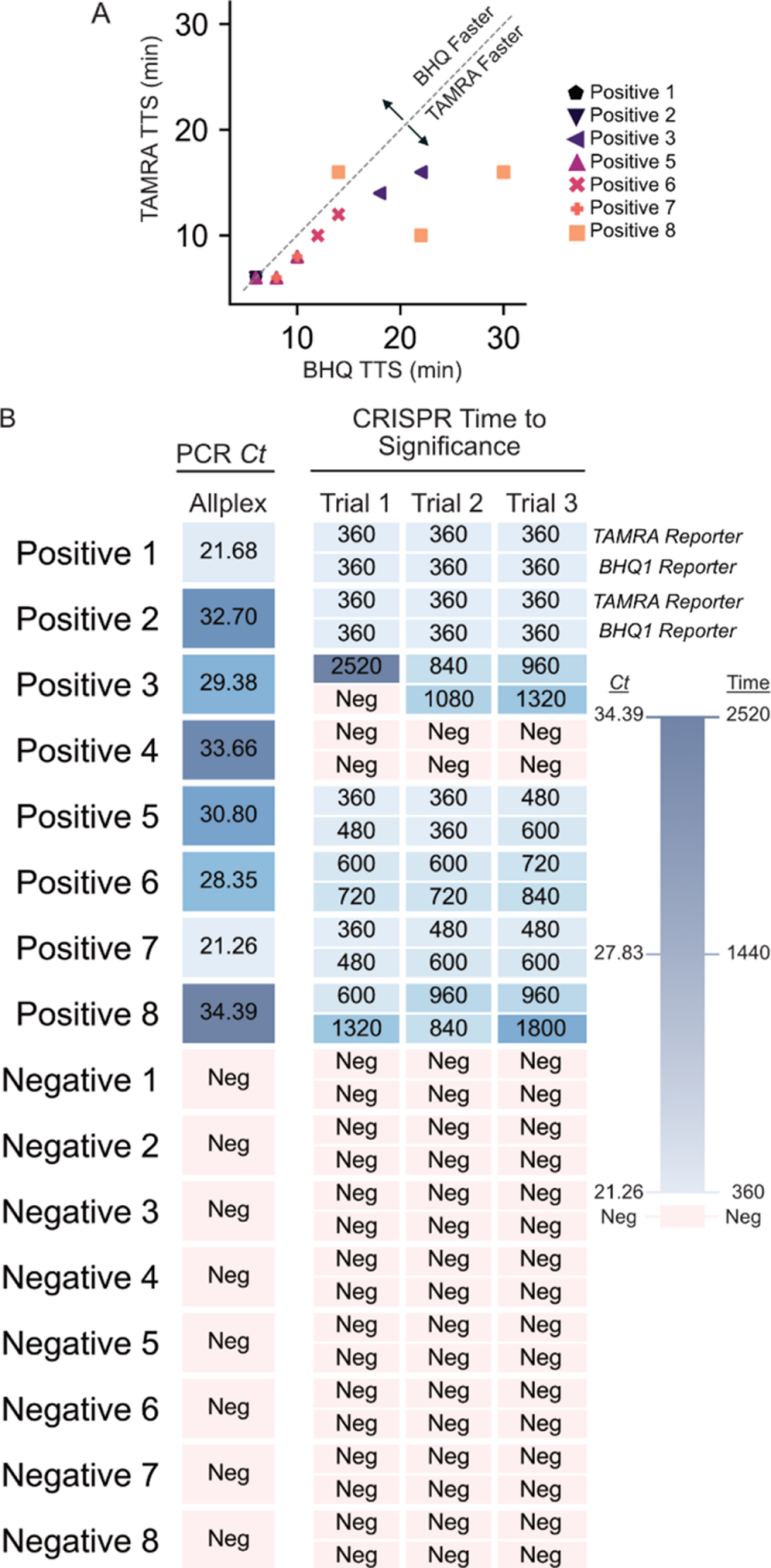
Detection of HPV-16 in patient-derived samples. (A) A comparison of test time-to-significance for the FAM–CCCCCC–BHQ-1 and FAM–CCCCCC–TAMRA reporters. The TAMRA reporter reaches significance consistently quicker than the BHQ1 reporter. Positive 4 and Positive 3 trial 1 are omitted from this analysis, as these did not reach significance during the assay. (B) A heatmap showing 8 positive clinical samples and 8 negative clinical samples. Each sample was analyzed in triplicate (trials 1 through 3) using both reporters. Each cell in the heatmap is labeled with either the *Ct* value (in the case of the leftmost PCR reference column) or the time-to-test significance in seconds (in the case of the CRISPR-Cas assays). The TAMRA reporter consistently reaches significance faster than the BHQ1 reporter, as well as catching one positive which the BHQ1 reporter missed (positive 3 trial 1). Further, all CRISPR-Cas assays failed to detect Positive 4.

Finally, we assessed the performance of the ratiometric reporter on patient-derived samples. Eight HPV-16-positive and eight HPV-16-negative (determined by qPCR, Table S2) cervical swab samples were analyzed in triplicate using an RPA–CRISPR-Cas12a-based assay (DETECTR assay).^7^ The conventional BHQ1-based reporter and the ratiometric reporter were both employed (Figures S6 and S7), and the time-to-significance data extracted (Figure 5A) and compared to the Allplex qPCR test (Figure 5B).

Data indicate that the ratiometric reporter is consistently faster than the BHQ1-based reporter. However, the time-to-significance decrease becomes more significant when assay times are longer. For example, samples such as positive sample 8 trials 1 and 3 took around half the time to achieve significance using the ratiometric measurement. Further, positive sample 3 trial 1 never reached a positive result using the BHQ1-based reporter but did eventually show a positive result using the ratiometric reporter. Neither reporter system detected any positive result in positive sample 4. We attribute this to the lower sensitivity of this NAAT–CRISPR-Cas assay when compared to the Allplex qPCR; the *Ct* value of this sample was 34, suggesting low target titers. While we did not observe a strong correlation between PCR Ct value and time-to-significance for either of the DETECTR assays, there was a strong relationship between samples testing positive with PCR and testing positive with the DETECTR assay. Thus, the false negative rates (percentage of positive samples that were not read as positive) of our assay were low (12.5 and ∼16.67% for the TAMRA and BHQ1-based reporters, respectively).

## Conclusions

This work demonstrates the benefits of employing visible dual-signal FRET-based reporters in CRISPR-Cas12a-based biosensors and highlights the ability to use them as “drop-in” substitutes for existing dark quench-based reporters. We show that the TAMRA-based reporter is more efficiently catalyzed by Cas12a when compared to a comparable BHQ1-based reporter and is more stable to DTT, a common additive in CRISPR-Cas12a-based assays.^7,34^ Building upon this, we have demonstrated that the TAMRA-based reporter affords a significant reduction in time-to-result in a model dual-sgRNA DETECTR assay and classical DETECTR assay performed on clinical samples.^6,31^

In addition to highlighting the clear benefits with regard to clinical sensitivity and time-to-result gained from switching to a dual-channel reporter, results unveil several interesting facts regarding the use of FRET-based reporters in CRISPR-Cas assays. First, the fact that Cas12a catalyzes the cleavage of the TAMRA-based reporter at a significantly faster rate than the BHQ1-based reporter suggests that terminal moieties on the ssDNA may influence the resulting shape of the reporter and, therefore, the capacity of the enzyme to cleave it. Second, this work highlights the benefits of ratiometric signal analysis for decreasing functional error and thus increasing assay robustness. Both of these discoveries have important implications for reporter engineering in CRISPR-Cas-based biosensors. However, a limitation of ratiometric signaling is the reliance on multiple excitation wavelengths and emission filters. While this technically necessitates a more complex instrument setup, the vast majority of benchtop readers are equipped as standard with several optical channels. Further, the usage of ratiometric readout can be enabled by adapting any of the multiple previously reported multichannel portable fluorescence readers.^35−38^

Developing new reporter systems to maximize signals in CRISPR-Cas sensors is essential in maximizing their impact. The current study highlights how relatively small changes in detection methodology or reporter structure can lead to dramatic benefits in assay performance. We believe there is significant scope to build upon this. For example, given the benefits of dual-channel ratiometric signaling, this work raises the question of whether adding additional signaling modalities (e.g., time-resolved fluorescence data) could be leveraged to improve the performance. Furthermore, it would be interesting to explore how other “drop-in” CRISPR-Cas reporters could benefit from dual-channel ratiometric signaling.^39−41^ Additional paths of inquiry could address the design considerations of optimizing quenched background vs the photostability of the cleaved fluorophore, with more stable fluorophores providing a less variable quotient and thus less noisy readout. Finally, elucidating the mechanisms behind how the cleavage rates of reporters change as a function of terminal moieties, for example, by using molecular modeling, could aid in designing a new generation of DETECTR reporters.

## Data Availability

All computer code, along with the specific package versions and computing environments used in this analysis, has been released at https://github.com/nkhosla/FRET_Ratiometric_CRISPR_Reporter.

## Supporting Information Available

Additional experiment protocols, details of all oligonucleotides used, statistical methods, additional supporting figures, specifically: assessment of reporter adsorption to the well plate, kinetic study of BHQ and TAMRA quenched reporters, data for individual patient-derived samples, and FAM–TAMRA FRET studies (PDF)

## Notes

**Ethical Statement**: This study was approved by the ethical committee of the University Hospital of Ghent (Belgium) under the reference 2019/1687. All participants provided written informed consent upon the start of the study for their samples to be used.

## Notes

We note that a preprint of this manuscript has been uploaded to ChemRxiv (10.26434/chemrxiv-2024-rrvb6). Stylistic changes have been made to align the manuscript more closely with the style of ACS Sensors and to address the previous reviewer’s comments. However, no significant changes have been made to the data, figures, or main content.
