# Supplementary material for: FRETting about CRISPR-Cas Assays: Dual-Channel Reporting Lowers Detection Limits and Times-to-Result
Source: ACS Sens. 2024 Jul 9;9(7):3616–24. doi: 10.1021/acssensors.4c00652 (PMC11287743; doi:10.1021/acssensors.4c00652)
Supplement: Supplementary file 1 — se4c00652_si_001.pdf [file se4c00652_si_001.pdf]

FRETting about CRISPR-Cas assays: Dual-Channel Reporting Lowers Detection Limits and Times-to-Result

Jake M. Lesinski[1]ǂ, Nathan K. Khosla[1]ǂ, Carolina Paganini[1], Bo Verberckmoes,[2] Heleen Vermandere,[2] Andrew J. deMello[1]*, and Daniel A. Richards[1]*

[1] Institute for Chemical and Bioengineering, ETH Zurich, Vladimir-Prelog-Weg 1, 8093 Zürich, Switzerland. [2] Faculty of Medicine and Health Sciences, Department of Public Health and Primary Care, Ghent University, De Pintelaan 185, 9000 Gent, Belgium

* Corresponding authors: daniel.richards@chem.ethz.ch, andrew.demello@chem.ethz.ch

ǂ Equal contribution.

Table of Contents

METHODS 2

OLIGONUCLEOTIDE DETAILS 2

EXPERIMENTAL PROTOCOLS 2

COMPUTATIONAL AND ANALYTICAL METHODS 4

TEST FOR SAMPLE NORMALITY 6

ASSESSMENT OF REPORTER ADSORPTION TO THE WELL PLATE 7

KINETIC STUDY OF BHQ AND TAMRA QUENCHED REPORTERS 7

COMPARING GUIDE CRRNAS 9

DATA FOR INDIVIDUAL CLINICAL SAMPLES 10

RATIOMETRIC READOUT AND ANALYSIS 10

SINGLE CHANNEL READOUT AT FAM EXCITATION AND EMISSION WAVELENGTHS 11

SELECTION OF FAM AND TAMRA AS A FRET PAIR 12

REFERENCES 13

Methods

Oligonucleotide details

Table S1. Sequences of the oligonucleotides used in this study. All oligonucleotides were commercially produced by Microsynth AG, Switzerland.

Oligo Name	Sequence (5' to 3')
**BHQ1-Quenched Reporter**	FAM - CCC CCC - BHQ1
**TAMRA-Quenched Reporter**	FAM - CCC CCC - TAMRA
**FAM-6C for Adsorption Studies**	FAM - CCC CCC
**TAMRA-6C for Adsorption Studies**	CCC CCC - TAMRA
**HPV16 crRNA guide 1**[1]	UAA UUU CUA CUA AGU GUA GAU UGA AGU AGA UAU GGC AGC AC
**HPV16 crRNA guide 2** [2]	UAA UUU CUA CUA AGU GUA GAU UUA CUG UUG UUG AUA CUA CA
**HPV16 RPA Primer Forward** [1]	TTG TTG GGG TAA CCA ACT ATT TGT TAC TGT T
**HPV16 RPA Primer Reverse** [1]	CCT CCC CAT GTC GTA GGT ACT CCT TAA AG

Experimental Protocols

Preparation of HOLMES Buffer

A 10x HOLMES buffer was prepared by combining the following reagents: 20 mM Spermidine, 400 mM Tris-HCl, 60 mM $MgCl_2$, 10mM DTT, 400 mM Glycine, 0.01% Triton X-100 (w/v), and 4% PEG-20,000 (w/v)[3,4]. Finally, the pH was adjusted to 8.5 with potassium acetate.

Characterizing Reporter Adsorption to the 384 Well Plate

To 10x HOLMES Buffer (2 µL), simulated cut reporter (FAM-CCCCCC or CCCCCC-TAMRA, or equal parts FAM-CCCCCC and CCCCCC-TAMRA) (1, 2, or 4 µL, 5 µM, UltraPure water) was added to make 250 nM, 500 nM and 1000 nM samples, respectively, and in quintuplet. All samples were then brought to a total volume of 20 µL using UltraPure water. Samples were then added to a 384 black well-plate (Corning, USA). Mineral oil (2.5 µL) was added to each well and the plate was centrifuged for 1 minute (1000 r.c.f.). The plate was then placed into a plate reader (Synergy H1, BioTek, USA) and the emission measured (Ex_{484}/Em_{530} and Em_{583}) every 2 minutes for 180 minutes.

Expression of LbCas12a Enzyme

The LbCas12a enzyme was expressed in *E. coli* BL21-GOLD (DE3) cells using an expression vector containing the DNA sequence for LbCas12a with an N-terminal 6xHis-tag and a C-terminal cysteine residue was (Twist Bioscience, USA). The cells were cultured in LB media at 37°C until an OD of 0.5 was reached. Next, protein expression was induced with 0.5 mM isopropyl D-thiogalactopyranoside (99%, PanReac AppliChem) and the culture was allowed to further grow for 20 hours at 20°C. The cells were harvested, resuspended in lysis buffer (50 mM Tris-HCl, 500 mM NaCl, 5% (v/v) glycerol, 1 mM TCEP, 0.5 mM PMSF, 10 mM imidazole, pH 7.5) and lysed by sonication. The recombinant protein in the soluble fraction of the lysate was isolated using immobilized metal ion affinity chromatography (Chelating Sepharose, Cytiva, USA) and then further purified by size exclusion chromatography

(HiLoad 16/600 Superdex 200 pg, Cytiva, USA) using a running buffer consisting of 20 mM Tris-HCl, 250 mM NaCl, 1mM TCEP, 5% (v/v) glycerol at pH 7.5. Finally, the protein was transferred into a storage buffer prepared in nuclease-free conditions (50 mM Tris-HCl, 500 mM NaCl, 5% (v/v) glycerol, 1 mM TCEP, pH 7.5) using an Amicon-15 centrifugal filter (50 kDa MWCO, RC membrane, Merck Millipore, Germany), concentrated, aliquoted and stored at -80°C.

CRISPR-Cas12a Double-Guide Assay with Synthetic Activating DNA

To two separate solutions of LbCas12a (217.5 µL, 115 nM, 1x HOLMES buffer), HPV-16 crRNA1 or HPV-16 cRNA2 (2.5 µL, 10 µM, UltraPure water) was added and the solution incubated at 37°C for 30 minutes to produce the two Cas–RNA complexes. These two complexes were mixed in equal parts, the mixture aliquoted (5 µL), and to each aliquot synthetic target DNA (1 µL, varying concentrations, UltraPure water), FAM–CCCCCC–BHQ-1 or FAM–CCCCCC–TAMRA reporter (0. 23 µL, 50 µM, UltraPure water), 10x HOLMES buffer (2.3 µL), and UltraPure water (14.47 µL) were added. These solutions were prepared in triplicate for each target DNA concentration. The samples were pipette mixed, then added to a chilled well 384 black well-plate (Corning, USA), which was kept on ice. Mineral oil (2.5 µL) was added to each well and the plate centrifuged for 1 minute (1000 r.c.f.). The plate was then placed into a plate reader (BioTek Synergy H1, USA) and the emission measured (Ex_{484}/Em_{530} and Em_{583}) every 2 minutes for 180 minutes.

Clinical Sample Collection

The samples were collected by a gynaecologist using a Viba brush (Viba Brush, Rovers, Oss, The Netherlands). The cervix and the superficial vaginal canal were swabbed with the brush, which then was rinsed in Hologic ThinPrep medium (Hologic Inc., Mississauga, ON, Canada).

Clinical Sample Processing

Cervical swabs were kept in Hologic ThinPrep medium and stored at 4-8°C, then concentrated and reconstituted in 200 µl PBS with 1% IGEPAL.

PCR Evaluation of Clinical Samples

Table S2. Clinical samples as analysed by Allplex, Anyplex, and Abott commercial testing platforms.

Sample	ALLPLEX	ANYPLEX	ABBOTT	ALLPLEX Final Result (*Ct* Value)
**1**	Positive for HPV16	16(+++)	Positive for HPV16	16 (21,63)
**2**	Positive for HPV16	16(++)	Positive for HPV16	16 (32,70)
**3**	Positive for HPV16	16(++)	Positive for HPV16	16 (29,38)
**4**	Positive for HPV16	16(++)	Positive for HPV16	16 (33,66)
**5**	Positive for HPV16	16(++)	Positive for HPV16	16 (30,80)
**6**	Positive for HPV16	16(++)	Positive for HPV16	16 (28,35)

**7**	Positive for HPV16	16(+++)	Positive for HPV16	16 (21,26)
**8**	Positive for HPV16	16(++)	Positive for HPV16	16 (34,39)

Computational and Analytical Methods

All computer code along with the specific package versions and computing environment used in this analysis has been released at https://github.com/nkhosla/FRET_Ratiometric_CRISPR_Reporter.

Propagation of Error

In various analyses, we divided random variables. Each variable had an associated standard error, and thus the error was propagated to the resulting quotient. However, as the variables were often in some way experimentally or physically related, we could not assume they were independent and had zero covariance. Accordingly, a full form of the approximation of propagated uncertainty was used[5,6] i.e.

$$f = \frac{X}{Y}$$

$$\sigma_f = \frac{X}{Y}\sqrt{\left(\frac{\sigma_X}{X}\right)^2 + \left(\frac{\sigma_Y}{Y}\right)^2 - 2\left(\frac{\sigma_{XY}}{XY}\right)}$$

Determination of Slope

Slope was calculated by combining all data points from a replicate series into one dataset and performing a linear regression from the initial point to time *t* to obtain the characteristic assay slope[7]. Specifically, the `linregress` function from the python `scipy.stats` package was used to obtain both the slope and the standard error of the calculated slope.

Time to Assay Significance Analysis

The time to assay significance, indicating the first time a statistically significant result could be read from the test, was defined as the first time, *t,* a given positive reached the following condition:

$$P(t) - 3\sigma_P > N(t) + 3\sigma_N$$

Where *P*(*t*) and *N*(*t*) are a positive test and its corresponding negative at time *t*, and σ_P and σ_N are their standard deviations, respectively.

Determination of Signal:Background Ratio

Signal to background was found by dividing any given positive by its corresponding negative. That is, the negative from the same test which was also analysed the same way (single channel, ratiometric, or slope analysis).

$$SBR(t) = \frac{P(t)}{N(t)}$$

It should be noted that any errors associated with the positive and negative readings were propagated as described above to calculate the SBR error.

Plotting CRISPR-Cas12a-Based Assay Data

All plots show a mean line (linearly interpolated between the data points) with shaded error ranges showing ±3 standard deviations. The shaded area is shown as a continuous region, but is also

interpolated between the data points. This representation of the data was used to enhance readability, as the alternative (plotting individual data points with mean markers and error bars) proved more difficult to interpret (**Figure S1**).

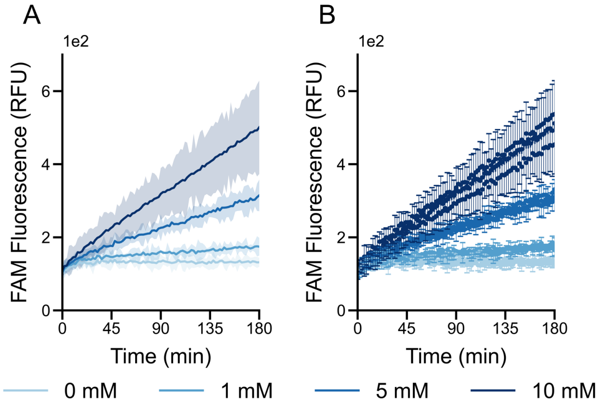

Figure S1. Comparison of plotting styles. We chose to emphasize readability by showing continuous lines, while noting our actual read rate in the methods for each experiment. (A) Our adopted plotting style, showing a mean line ±3 standard deviations, both interpolated between data points. (B) The alternative, showing the same data with individual data points and error bars, along with a small mean marker. The lack of transparency makes overlapping bars hard to read, whereas adding transparency would make the thin lines hard to read.

Test for Sample Normality

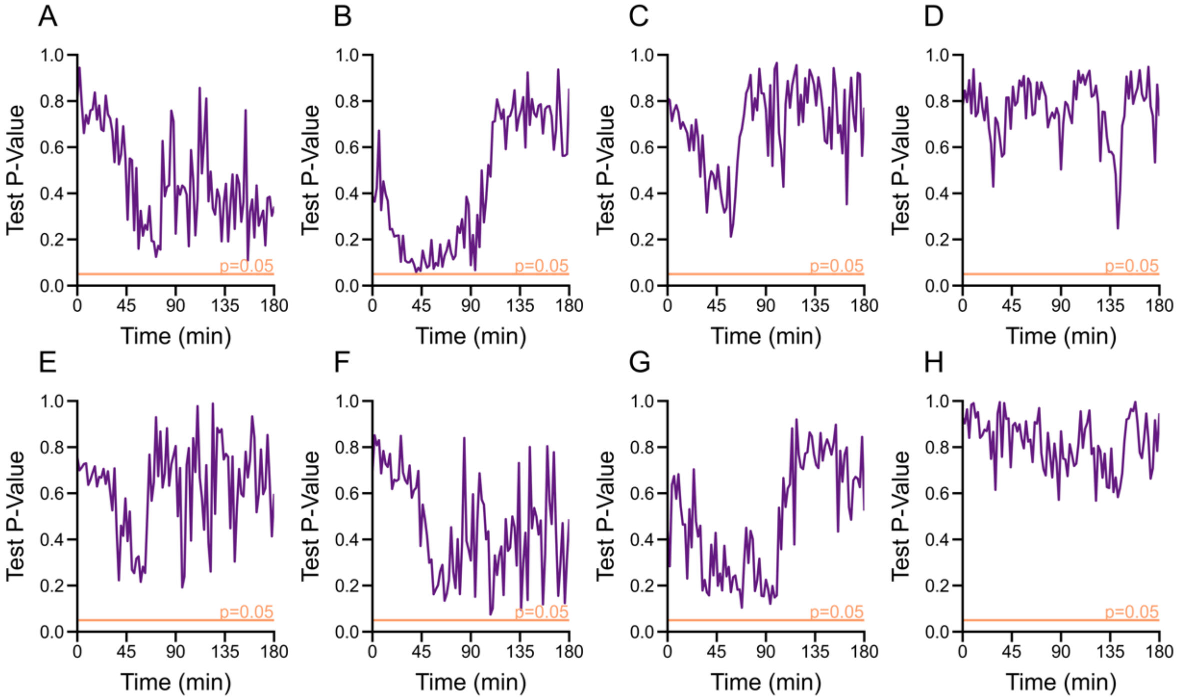

Figure S2. Normality testing. To properly propagate uncertainty though the ratiometric analysis of the signals from FAM–CCCCCC–TAMRA, we assumed that our readings (n=20) at each time were distributed normally. To validate this assumption we used the normality test based on the method by D'Agostino and Pearson (taking into account skew and kurtosis) as implemented in the *stats.normaltest* function in the *scipy* python package[8–10] (A) FAM channel negative tests. (B) FAM channel 2 pM tests. (C) FAM channel 20 pM tests. (D) FAM channel 200 pM tests. (E) TAMRA channel negative tests. (F) TAMRA channel 2 pM tests. (G) TAMRA channel 20 pM tests. (H) TAMRA channel 200 pM tests. The FAM channel is defined as Ex_{484}/Em_{530}. The TAMRA channel is defined as Ex_{484}/Em_{583}.

Kinetic Study of BHQ and TAMRA Quenched Reporters

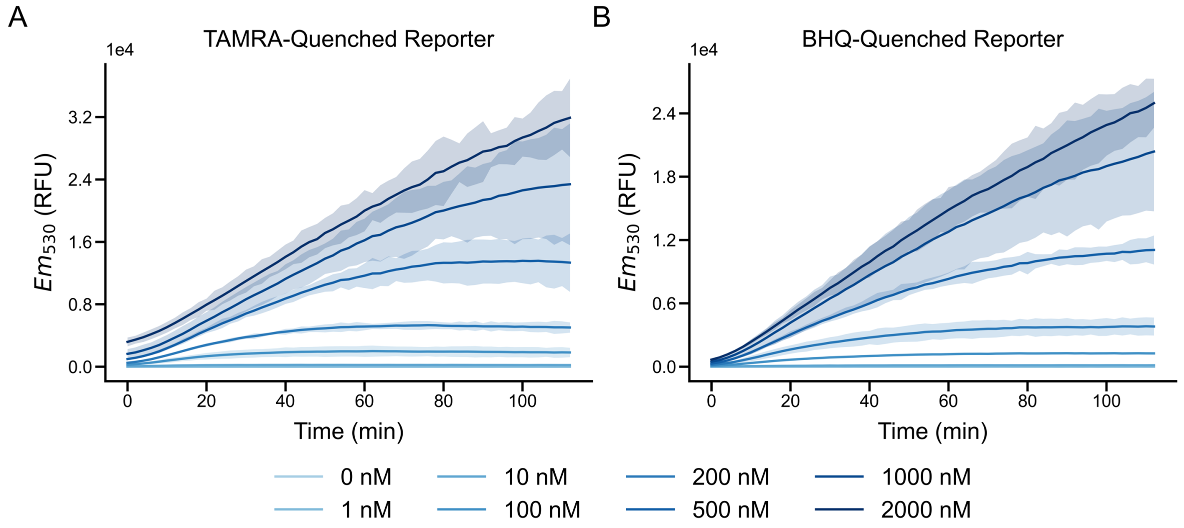

Figure S3. Kinetic tests of the Cas12a enzyme with both guide crRNAs at various reporter concentrations. (A) Cas12a + crRNA 1 complex tested at 0 nM, 1 nM, 10 nM, 100 nM, 200 nM, 500 nM, 1000 nM, and 2000 nM of FAM-CCCCCC–TAMRA reporter. (B) Cas12a + crRNA 1 complex tested at 0 nM, 1 nM, 10 nM, 100 nM, 200 nM, 500 nM, 1000 nM, and 2000 nM of FAM-BHQ1 reporter. For both analyses, the signal was obtained from a single channel (Ex_{484}/Em_{530}) with readings every two minutes. All analyses are plotted as the mean of three replicates (line) ± three standard deviations (shaded region).

Assessment of Reporter Adsorption to the Well plate

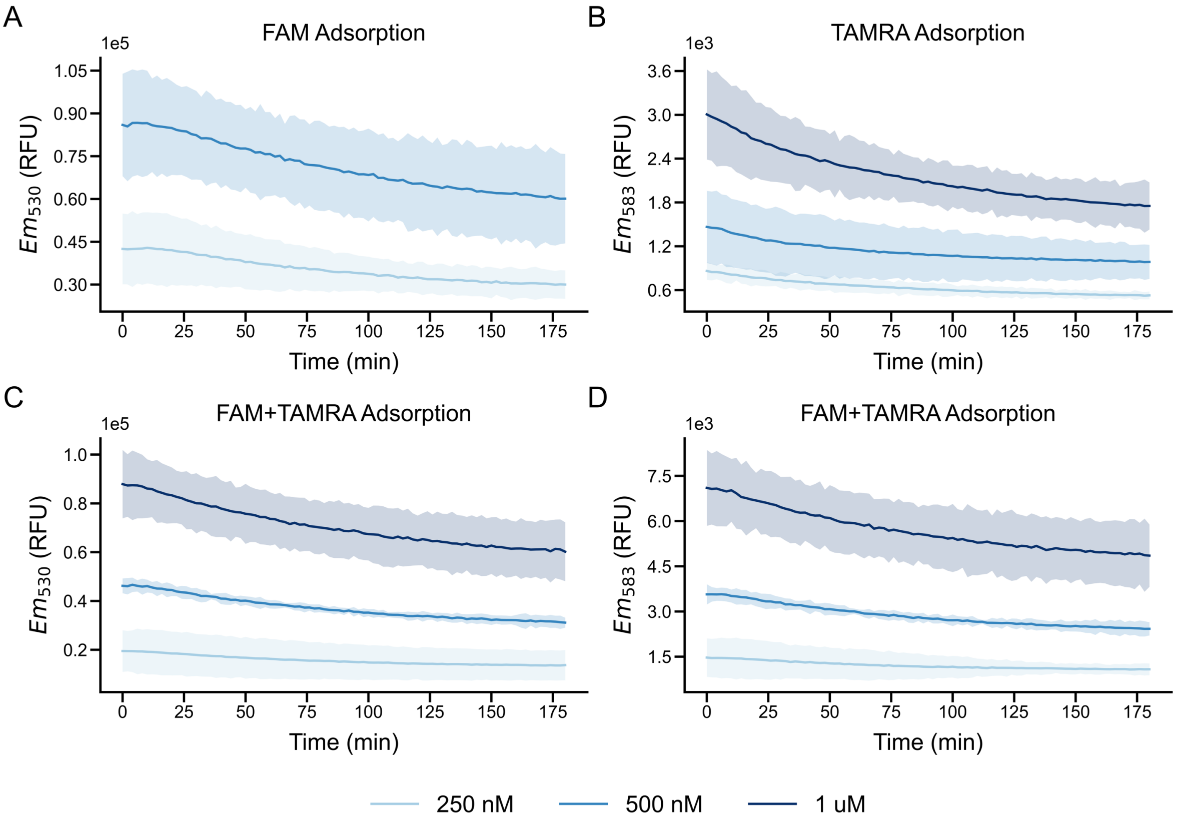

Figure S4. Assessment of adsorption of FAM-DNA and TAMRA-DNA to wells of a 384-well plate. (A) 250 nM, 500 nM, and 1 uM of FAM-CCCCCC DNA in HOLMES Buffer. (B) 250 nM, 500 nM, and 1 uM of TAMRA-CCCCCC DNA in HOLMES Buffer. (C and D) 250 nM, 500 nM, and 1 uM of equal parts FAM-CCCCCC and TAMRA-CCCCCC DNA in HOLMES Buffer. For each assay, 20 μl of each sample was placed in a well plate (in quintuplet) and observed over a period of three hours. Measurements were taken every two minutes. Readouts were taken at both Ex_{484}/Em_{530} (C) and Ex_{484}/Em_{583} (D). All analyses are plotted as the mean of five replicates (line) ± three standard deviations (shaded region).

Comparing Guide crRNAs

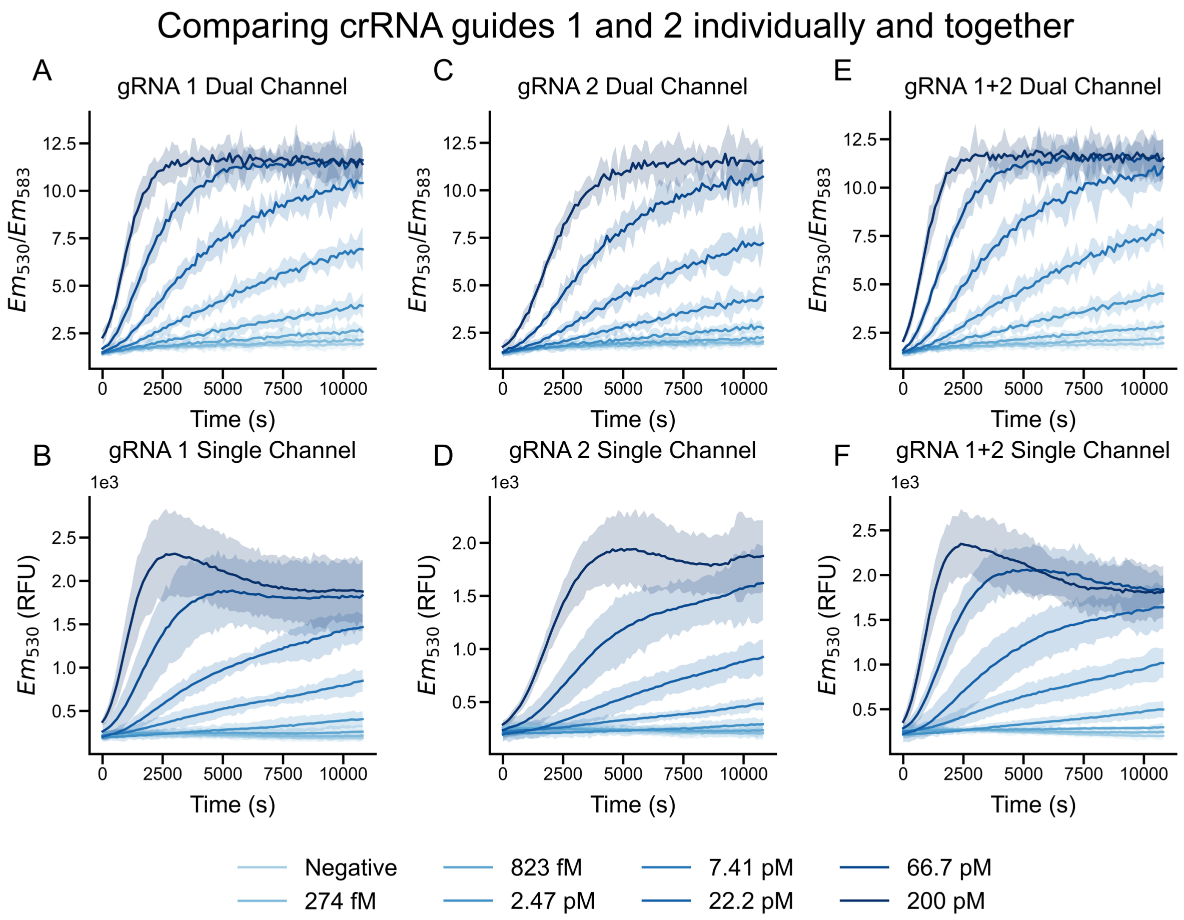

Figure S5. Comparison of the two guide crRNAs, individually and together. The guide cRNAs were incubated with varying concentrations of target DNA and are plotted as follows: (A) cRNA 1 with two channel readout; (B) cRNA1 with single channel readout; (C) cRNA 2 with two channel readout; (D) cRNA 2 with single channel readout; (E) cRNA 1 + cRNA 2 with two channel readout; (F) cRNA 1 + cRNA 2 with single channel readout. For two channel readout, measurements were taken at both Ex_{484}/Em_{530} and Ex_{484}/Em_{584}, and the ratio is plotted. For single channel readout, measurements were taken at Ex_{484}/Em_{530} and Ex_{484}/Em_{584}, and the relative fluorescence plotted. All analyses are plotted as the mean of three replicates (line) ± three standard deviations (shaded region).

Data For Individual Clinical Samples

Ratiometric readout and analysis

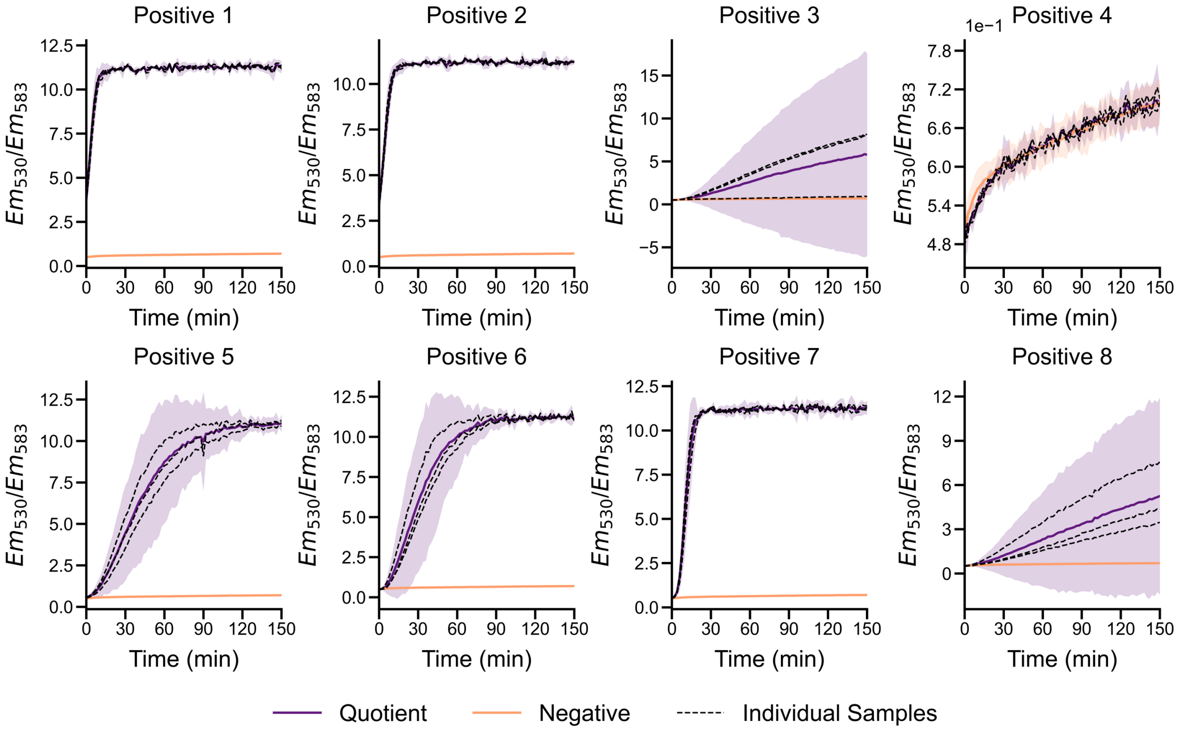

Figure S6. Ratiometric readout and analysis of positive clinical samples. Dual channel (Ex_{484}/Em_{530} and Ex_{484}/Em_{584}) readout of all eight clinical samples (tested using a FAM-CCCCCC-TAMRA reporter), showing the sample average (line) ± three standard deviations (shaded region). Each positive is plotted in comparison to the mean of all negatives (eight different clinical samples, three tests per sample) ± three standard deviations of the negative data.

Single channel readout at FAM excitation and emission wavelengths

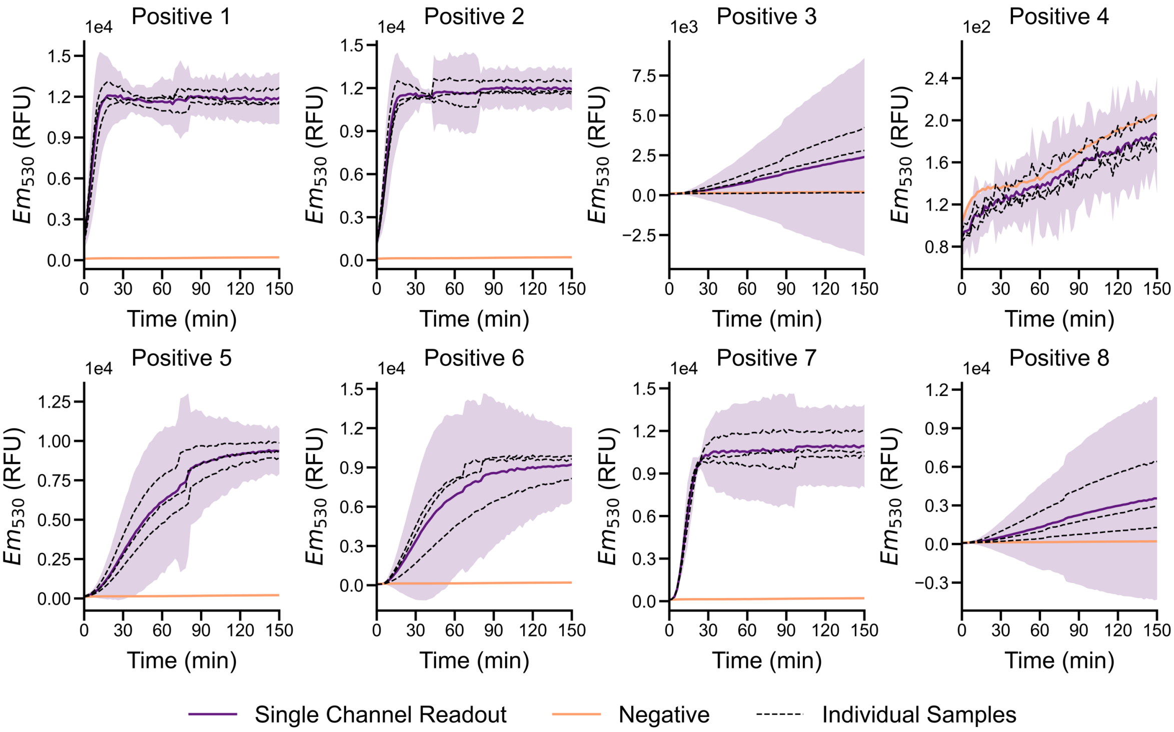

Figure S7. Single channel readout and analysis of positive clinical samples. Single channel (Ex_{484}/Em_{530}) readout of all eight clinical samples (tested using a FAM-CCCCCC-BHQ reporter), showing the sample average (line) ± three standard deviations (shaded region). Each positive is plotted in comparison to the mean of all negatives (8 different clinical samples, 3 tests per sample) ± three standard deviations of the negative data.

Selection of FAM and TAMRA as a FRET pair

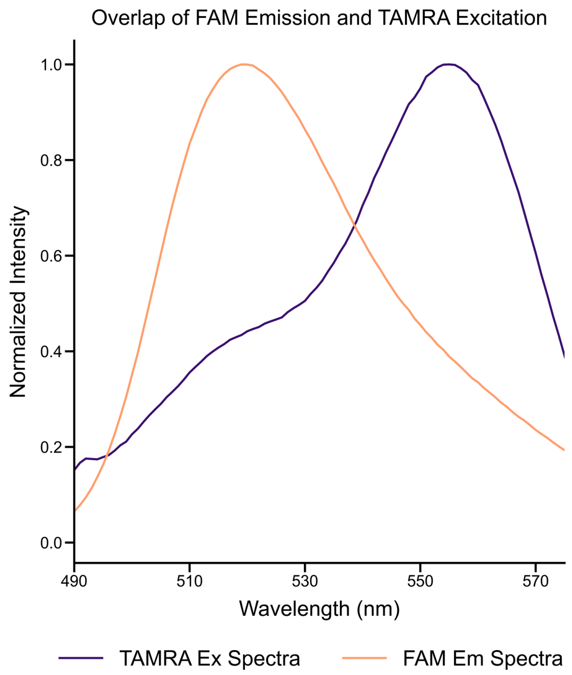

Figure S8. Overlap of FAM Emission with TAMRA Excitation. To choose a FRET pair we optimized the spectral overlap of the FRET donor and acceptor. The FAM emission spectrum was taken by exciting at 490 nm. The TAMRA excitation spectrum was measured with an emission wavelength of 583 nm. Both of these wavelengths were chosen to match the wavelengths used in the CRISPR-Cas assay using a plate reader.

References

(1) Chen, J. S.; Ma, E.; Harrington, L. B.; Da Costa, M.; Tian, X.; Palefsky, J. M.; Doudna, J. A. CRISPR-Cas12a Target Binding Unleashes Indiscriminate Single-Stranded DNase Activity. *Science* **2018**, *360* (6387), 436–439.

(2) Gong, J.; Zhang, G.; Wang, W.; Liang, L.; Li, Q.; Liu, M.; Xue, L.; Tang, G. A Simple and Rapid Diagnostic Method for 13 Types of High-Risk Human Papillomavirus (HR-HPV) Detection Using CRISPR-Cas12a Technology. *Sci Rep* **2021**, *11*, 12800.

(3) Lv, H.; Wang, J.; Zhang, J.; Chen, Y.; Yin, L.; Jin, D.; Gu, D.; Zhao, H.; Xu, Y.; Wang, J. Definition of CRISPR Cas12a Trans-Cleavage Units to Facilitate CRISPR Diagnostics. *Frontiers in Microbiology* **2021**, *12*, 766474.

(4) Li, S.-Y.; Cheng, Q.-X.; Wang, J.-M.; Li, X.-Y.; Zhang, Z.-L.; Gao, S.; Cao, R.-B.; Zhao, G.-P.; Wang, J. CRISPR-Cas12a-Assisted Nucleic Acid Detection. *Cell Discov* **2018**, *4*, 20.

(5) Ku, H. H. Notes on the Use of Propagation of Error Formulas. *Journal of Research of the National Bureau of Standards C. Engineering and Instrumentation* **1966**, *70C* (4), 263–273.

(6) Tellinghuisen, J. Statistical Error Propagation. *J. Phys. Chem. A* **2001**, *105* (15), 3917–3921.

(7) Fozouni, P.; Son, S.; Derby, M. D. de L.; Knott, G. J.; Gray, C. N.; D'Ambrosio, M. V.; Zhao, C.; Switz, N. A.; Kumar, G. R.; Stephens, S. I.; Boehm, D.; Tsou, C.-L.; Shu, J.; Bhuiya, A.; Armstrong, M.; Harris, A. R.; Chen, P.-Y.; Osterloh, J. M.; Meyer-Franke, A.; Joehnk, B.; Walcott, K.; Sil, A.; Langelier, C.; Pollard, K. S.; Crawford, E. D.; Puschnik, A. S.; Phelps, M.; Kistler, A.; DeRisi, J. L.; Doudna, J. A.; Fletcher, D. A.; Ott, M. Amplification-Free Detection of SARS-CoV-2 with CRISPR-Cas13a and Mobile Phone Microscopy. *Cell* **2021**, *184* (2), 323-333.

(8) Virtanen, P.; Gommers, R.; Oliphant, T. E.; Haberland, M.; Reddy, T.; Cournapeau, D.; Burovski, E.; Peterson, P.; Weckesser, W.; Bright, J.; van der Walt, S. J.; Brett, M.; Wilson, J.; Millman, K. J.; Mayorov, N.; Nelson, A. R. J.; Jones, E.; Kern, R.; Larson, E.; Carey, C. J.; Polat, İ.; Feng, Y.; Moore, E. W.; VanderPlas, J.; Laxalde, D.; Perktold, J.; Cimrman, R.; Henriksen, I.; Quintero, E. A.; Harris, C. R.; Archibald, A. M.; Ribeiro, A. H.; Pedregosa, F.; van Mulbregt, P. SciPy 1.0: Fundamental Algorithms for Scientific Computing in Python. *Nat Methods* **2020**, *17* (3), 261–272.

(9) D'Agostino, R. B. An Omnibus Test of Normality for Moderate and Large Size Samples. *Biometrika* **1971**, *58* (2), 341–348.

(10) D'Agostino, R.; Pearson, E. S. Tests for Departure from Normality. Empirical Results for the Distributions of B2 and √ B1. *Biometrika* **1973**, *60* (3), 613–622.
